# Performance of a flow cytometry-based immunoassay for detection of antibodies binding to SARS-CoV-2 spike protein

**DOI:** 10.1038/s41598-021-04565-1

**Published:** 2022-01-12

**Authors:** Arantxa Valdivia, Fabián Tarín, María Jesús Alcaraz, Paula Piñero, Ignacio Torres, Francisco Marco, Eliseo Albert, David Navarro

**Affiliations:** 1 Microbiology Service, Clinic University Hospital, INCLIVA Health Research Institute, Valencia, Spain; 2 Hematology Department, Hospital General Universitario Alicante, Alicante, Spain; 3 Immunology Department, Hospital General Universitario Alicante, Alicante, Spain; 4 Department of Microbiology, School of Medicine, University of Valencia, Av. Blasco Ibáñez 17, 46010 Valencia, Spain

**Keywords:** Infectious diseases, Microbiology, Infectious-disease diagnostics, Virology

## Abstract

The performance of a laboratory-developed IgG/IgA flow cytometry-based immunoassay (FCI) using Jurkat T cells stably expressing full-length native S protein was compared against Elecsys electrochemiluminiscent (ECLIA) Anti-SARS-CoV-2 S (Roche Diagnostics, Pleasanton, CA, USA), and Liaison SARS-CoV-2 TrimericS IgG chemiluminiscent assay (CLIA) (Diasorin S.p.a, Saluggia, IT) for detection of SARS-CoV-2-specific antibodies. A total of 225 serum/plasma specimens from 120 acute or convalescent COVID-19 individuals were included. Overall, IgG/IgA-FCI yielded the highest number of positives (n = 179), followed by IgA-FCI (n = 177), Roche ECLIA (n = 175), IgG-FCI (n = 172) and Diasorin CLIA (n = 154). For sera collected early after the onset of symptoms (within 15 days) IgG/IgA-FCI also returned the highest number of positive results (52/72; 72.2%). Positive percent agreement between FCI and compared immunoassays was highest for Roche ECLIA, ranging from 96.1 (IgG/IgA-FCI) to 97.7% (IgG-FCI), whereas negative percent agreement was higher between FCI and Diasosin CLIA, regardless of antibody isotype. The data suggest that FCI may outperform Roche ECLIA and Diasorin CLIA in terms of clinical sensitivity for serological diagnosis of SARS-CoV-2 infection.

## Introduction

SARS-CoV-2 serological assays enable us to identify individuals infected either recently or in the past, assess humoral immune responses elicited by SARS-CoV-2 vaccines and infer level of susceptibility to reinfection or primary infection in vaccinees^1,2^. Among SARS-CoV-2 structural components, Spike protein (S) elicits the most potent neutralizing antibodies, which are crucially involved in protecting against SARS-CoV-2 infection^3,4^. The S protein, which is assembled into trimers in the viral membrane, interacts with angiotensin converting enzyme type 2 receptor (ACE2) through the receptor-binding domain (RBD)^5^. Binding of RBD to ACE2 promotes cleavage of S into S1 and S2, and exposure of the fusion peptide located within S2, which eventually leads to cellular and viral membrane fusion^5^. A large number of immunoassays using recombinant RBD, S1 or S2 subunits or full-length monomeric S protein as the binding antigen and returning either qualitative or semiquantitative results have been developed, evaluated in different studies and found to exhibit variable sensitivity and specificity [see Refs.^1,2,6,7^ for review]. A new generation of recently launched commercially-available SARS-CoV-2 immunoassays detect antibodies binding to the SARS-CoV-2 S protein in its native (trimeric) conformation or RBD and offer quantitative estimates of antibody levels^8–11^, and preliminary results show increased sensitivity for detection of SARS-CoV-2 antibodies, as well as reliable estimates of serum neutralizing activity against SARS-CoV-2. In this context, a flow cytometry-based immunoassay (FCI) has been developed employing Jurkat T cells stably expressing the full-length native S protein, which is reported to be highly specific and display greater sensitivity than various comparative immunoassays targeting recombinant RBD or S subunit proteins^12,13^. Here, we evaluated the performance of this FCI against two new-generation immunoassays: Elecsys Anti-SARS-CoV-2 S (Roche Diagnostics, Pleasanton, CA, USA), and Liaison SARS-CoV-2 TrimericS IgG assay (Diasorin S.p.a, Saluggia, IT), using sera from in- or outpatients with SARS-CoV-2 infection documented by RT-PCR.

## Patients and methods

### Patients and specimens

The current retrospective study was carried out using cryopreserved (− 20 °C) serum or plasma samples collected from the following four groups: (I) Convalescent COVID-19 patients, as clinically^14^ and microbiologically documented by RT-PCR^15^, who had been admitted to different hospital wards and eventually released. A total of 60 specimens from 35 patients, drawn at a median of 60 days (range 8–141 days) since symptoms onset were included; (II) Acute COVID-19 patients admitted to the intensive care unit (ICU). A total of 115 specimens from 40 patients, collected at a median of 16 days (range 2–43 days) after onset of symptoms were included; (III) Acute or convalescent COVID-19 subjects (n = 45) who tested negative by rapid lateral flow immunoassay-LFIC- (Innovita 2019‐nCoV Ab Test; Beijing Innovita Biological Technology, China), or CLIA (Liaison SARS-CoV-2 S1/S2 IgG CLIA; DiaSorin, Saluggia, Italy, the Maglumi 2019-nCoV IgG SNIBE—Shenzhen New Industries Biomedical Engineering Co., Ltd, Shenzhen, China, or both) in use in our laboratory at the time of sample collection and routine testing. A total 50 specimens from this group, collected at a median of 44 days after onset of symptoms (range 11–91) were included, of which 13 specimens tested negative by LFIC, 34 returned negative results by Liaison assay and 7 by Maglumi assay. Thus, a total 225 sera were available from these three groups for analyses. All these sera were tested by the FCI assay, whereas due to sample volume constraints, 217 and 215 were run in the Roche and Diasorin platforms, respectively. (IV) Pre-pandemic sera obtained from unique blood donors (n = 100). Specimens belonging to different groups of SARS-CoV-2-infected individuals were combined or treated individually, as appropriate for study purposes. The study was approved by the Ethics Committee of Hospital Clínico Universitario INCLIVA, all methods were performed in accordance with the relevant guidelines and regulations.

### Flow cytometry native SARS-CoV-2 S assay

We measured IgG and IgA antibody levels by FCI as previously described in detail^12,13^. Previously published data^12^ found detection of SARS-CoV-2-S-binding IgM to be less consistent and reliable. These analyses were carried out at the Hematology Department of Hospital General Universitario, Alicante, Spain. Briefly**,** transfected human Jurkat T-cell line (clone E6-1) stably expressing both the full-length native SARS-CoV-2 S protein and a truncated version of the human Epidermal growth factor receptor (huEGFRt) were used as the binding antigens (S-Jurkat). Non-transfected Jurkat cells (0-Jutkat) were used as controls. For each individual assay, a mixture of 50,000 0-Jurkat and 150,000 S-Jurkat cells was made in a single tube. Sera were diluted 1:50 in phosphate-buffered saline (PBS) containing 1% bovine serum albumin (BSA) and 0.02% sodium azide and incubated with the cell mixture for 30 min on ice. The cells were then spun down, washed with PBS-BSA and stained with mouse anti-human IgG-PerCP Jackson ImmunoResearch¸ Cambridgeshire, UK), anti-human IgA-Alexa Fluor 647 (Jackson ImmunoResearch) and anti-human EGFR (BV421) (Biolegend, San Diego, CA, USA). Samples were then washed and acquired on an Omnicyt flow cytometer (Cytognos S.L, Salamanca, Spain) and analyzed using the Infinicyt 2.0 software (Cytognos SL). Flow cytometer MFI target values were established in the 5th peak of Rainbow beads (Cytognos SL), according to manufacturer’s instructions^13^. Particle data was acquired in each instrument run. The gating strategy has been previously detailed^13^. A minimum of 50,000 viable events, discarding doublets and debris, were considered for the analyses. IgG or IgA antibodies bound to S proteins were identified by comparing the median fluorescence intensity (MFI) of the S-Jurkat and the 0-Jurkat cells in each sample. We established the difference between S-Jurkat and 0-Jurkat cells using the normalized MFI-ratio between EGFR and both antibody isotypes (IgG MFI-ratio and IgA MFI-ratio respectively), calculated as follows: (IgG/IgA MFI of S-Jurkat—IgG/IgA MFI of 0-Jurkat)/(EGFR MFI of S-Jurkat—EGFR MFI of 0-Jurkat). Samples were considered positive for IgG or IgA when the normalized difference was ≥ 1, as all pre-pandemic sera yielded IgG and IgA-MFI ratios below 1 (mean, 0.55, SD, 0.31 and 0,71, SD, 0.24, respectively). For qualitative IgG and IgA results (positive vs. negative) the inter-assay rate of agreement was 100%.

### Commercially-available chemiluminescent SARS-CoV-2 S assays

Roche Elecsys Anti-SARS-CoV-2 S (Roche Diagnostics, Pleasanton, CA, USA), an electrochemiluminescence sandwich immunoassay (ECLIA) that quantifies total (IgG and IgM) antibodies directed against RBD, was run on cobas e601 modular analyzer (Roche Diagnostics, Rotkreuz, Switzerland). The assay is calibrated with the first WHO International Standard and Reference Panel for anti-SARS-CoV-2 antibody^16^. The limit of detection of the assay is 0.4 U/ml and its quantification range is between 0.8 and 250.0 U/mL. The Liaison SARS-CoV-2 TrimericS IgG assay (Diasorin S.p.a, Saluggia, Italy), run on a DiaSorin Liaison platform (DiaSorin, Stillwater, USA), measured IgG antibodies against a trimeric S-protein antigen. Samples yielding < 13 AU/mL were considered negative. According to the manufacturer, the upper quantification limit of the assay is 800 AU/mL. Specimens yielding values above the upper quantification limit of the respective assay were conveniently diluted (up to 1/10, to maintain linearity, according to the manufacturers) and re-assayed. Intra and inter-assay coefficient of variation of these assays are < 5%, according to the respective manufacturer. Both immunoassays were performed at the Microbiology Service at the Hospital Clínico Universitario, Valencia, Spain, following the instructions of the respective manufacturers.

#### Statistical methods

Positive and negative percent agreement (PPA and NPA, respectively) between immunoassays were calculated using a diagnostic 2 × 2 test. Cohen’s Kappa statistics was used to assess the degree of concordance between qualitative results provided by the immunoassays and interpreted as previously recommended^17^. Two-sided exact *P* values were reported. A *P* value < 0.05 was considered statistically significant. The analyses were performed using SPSS version 20.0 (SPSS, Chicago, IL, USA).

### Ethical statement

The current study was approved by the Research Ethics Committee of Hospital Clínico Universitario INCLIVA (September, 2019). As it was a retrospective analysis, the ethics committee exempted us from obtaining the informed consent of the patients.

## Results

When combining specimens from all three groups of SARS-CoV-2-infected patients, we found that IgG/IgA-FCI yielded the highest number of positives (n = 179), closely followed by IgA-FCI (n = 177), Roche ECLIA (n = 175), and IgG-FCI (n = 172) (Table 1). Diasorin CLIA returned a substantially lower number of positive results (n = 154) than the former platforms. A subanalysis was next conducted including only sera (n = 50) that scored negative by LFIC or CLIA assays routinely used at our laboratory at the time of testing request. As shown in Table 2, FCI (either IgG, IgA or IgG/IgA) yielded a greater number of positive results than Roche ECLIA or Diasorin CLIA.

**Table 1:** Performance comparison between a flow cytometry-based S immunoassay and two commercially-available SARS-CoV-2 chemiluminescent immunoassays.

Assays under comparison (no. of specimens)	Flow cytometry SARS-CoV-2 S assay result (IgG/IgA/IgG + IgA)		Liaison^®^ SARS-CoV-2 TrimericS IgG assay	
	Positive	Negative	Positive	Negative
Positive by Roche Elecsys^®^ Anti-SARS-CoV-2 S (175)	168/170/172	7/5/3	151	17
Negative by Roche Elecsys^®^ Anti-SARS-CoV-2 S (42)	4/7/7	38/35/35	1	41
Positive by Liaison^®^ SARS-CoV-2 TrimericS IgG assay (154)	150/152/153	4/2/1	–	–
Negative by Liaison^®^ SARS-CoV-2 TrimericS IgG assay (61)	20/23/24	41/38/37	–	–

*RBD* receptor binding domain, *S* spike protein.

**Table 2:** Qualitative results returned by Flow cytometry-based and CLIA assay in specimens from RT-PCR-confirmed SARS-CoV-2 infection testing negative by immunoassays in use at the time of specimen collection.

Result by corresponding assay	Flow cytometry SARS-CoV-2 S assay (IgG/IgA/IgG + IgA)	Roche Elecsys^®^ anti-SARS-CoV-2 S^a^	Liaison^®^ SARS-CoV-2 TrimericS IgG assay^a^
Positive	18/22/22	13	5
Negative	32/28/28	34	43

Either lateral flow immunochromatography assay (INNOVITA 2019‐nCoV Ab Test; Beijing Innovita Biological Technology, China), LIAISON SARS-CoV-2 S1/S2 IgG chemiluminescent assay (DiaSorin S.p.A., Saluggia, Italy) or MAGLUMI 2019-nCoV IgG assay (SNIBE—Shenzhen New Industries Biomedical Engineering Co., Ltd, Shenzhen, China).

^a^2 and 3 specimens could not be analyzed by Liaison and Roche assay, respectively, due to insufficient sample volume.

We next compared the performance of the three immunoassays across different arbitrarily defined time frames of sample collection since the onset of COVID-19 symptoms: within 15 days, between 16 and 30 days and more than 30 days. The data are shown in Table 3. FCI assay returned the highest number of positive results in sera collected early after the onset of symptoms (within 15 days) followed by the Roche and Diasorin assays. For sera obtained afterwards, the Roche assay yielded higher number of positive results followed by FCI and Diasorin CLIA.

**Table 3:** Performance of a flow cytometry-based S immunoassay and two commercially-available SARS-CoV-2 CLIA targeting either the trimeric S protein or the receptor binding domain (RBD) according to the time of sample collection since the onset of COVID-19 symptoms.

Immunoassay	Time of sera collection since the onset of COVID-19		
	< 15 days Positive/negative results (% positives)	16–29 days Positive/negative results (% positives)	≥ 30 days Positive/negative results (% positives)
Flow cytometry SARS-CoV-2 S assay result (IgG)	50/22 (69.4)	45/12 (78.9)	77/19 (80.2)
Flow cytometry SARS-CoV-2 S assay result (IgA)	51/21 (70.8)	43/14 (75.4)	83/13 (86.4)
Flow cytometry SARS-CoV-2 S assay result (IgG + IgA)	52/20 (72.2)	44/13 (77.1)	83/13 (86.4)
Roche Elecsys^®^ anti-SARS-CoV-2 S	46/25 (64.7)	48/19 (84.2)	81/8 (91.0)
Liaison^®^ SARS-CoV-2 TrimericS IgG assay	44/27 (61.9)	37/15 (71.1)	73/19 (79.3)

Overall, PPA between FCI and the immunoassay compared was highest for Roche ECLIA, ranging from 96.1% (IgG/IgA-FCI) to 97.7% (IgG-FCI) (Table 4), whereas NPA was overall greater between FCI and Diasosin CLIA, regardless of the antibody isotype detected (91.4% to 97.2%). Inter-rater agreement between FCI (either IgG, IgA or IgG/IgA) and Roche ECLIA was strong (*k* ≥ 0.8), while it was only moderate with Diasorin CLIA (*k* ≥ 0.6 to < 0.8). Inter-rater agreement between results returned by Roche ECLIA and Diasorin CLIA was also moderate (*k* = 0.76).

**Table 4:** Agreement between qualitative results provided by flow cytometry-based S immunoassay and two commercially-available SARS-CoV-2 CLIA targeting either the trimeric S protein or the receptor binding domain (RBD).

Assays under comparison	Percent agreement between qualitative results (95% CI)		Kappa index (*P* value)
	Positive	Negative	
IgG FCI/Roche Elecsys^®^ anti-SARS-CoV-2 S	97.7 (95.4–99.9)	84.4 (73.8–95.0)	0.84 (< 0.001)
IgA FCI/Roche Elecsys^®^ anti-SARS-CoV-2 S	96.0 (93.1–98.9)	87.5 (77.2–97.7)	0.82 (< 0.001)
IgG + IgA FCI/Roche Elecsys^®^ anti-SARS-CoV-2 S	96.1 (92.2–98.9)	92.1 (83.5–100)	0.85 (< 0.001)
IgG FCI/Liaison^®^ SARS-CoV-2 TrimericS IgG assay	88.2 (83.3–93.1)	91.1 (82.8–99.4)	0.69 (< 0.001)
IgA FCI/Liaison^®^ SARS-CoV-2 TrimericS IgG assay	86.8 (81.8–91.8)	95.0 (88.25–100)	0.68 (< 0.001)
IgG + IgA FCI/Liaison^®^ SARS-CoV-2 TrimericS IgG assay	86.4 (81.4–91.5)	97.4 (92.3–100)	0.68 (< 0.001)
Roche Elecsys^®^ Anti-SARS-CoV-2 S/Liaison^®^ SARS-CoV-2 TrimericS IgG assay	89.8 (85.3–94.4)	97.6 (93.0–100)	0.76 (< 0.001)

*FCI* flow cytometry immunoassay.

## Discussion

In this study we compared the performance of an in-house-developed quantitative FCI^12,13^ with the SARS-CoV-2 trimericS-IgG CLIA from Diasorin and Roche RBD-specific IgG/IgM antibody ECLIA for serological diagnosis of SARS-CoV-2 infection in patients with either acute or convalescent COVID-19. The latter two have been reported to measure serum/plasma antibody levels that correlate with those quantified by virus neutralization assays, using either wild type SARS-CoV-2 or lentiviral-S-pseudotyped virions^8–11^. Of note, only Roche ECLIA is calibrated to the first WHO International Standard and Reference Panel for anti-SARS-CoV-2 antibody (15). By using a large number of pre-pandemic sera we set up an MFI ratio (≥ 1) yielding maximum specificity. A previous report also found the FCI assay to provide an specificity of 100%^12^. Nevertheless, it must be stressed that we are not certain that sera from individuals with past seasonal coronavirus infection were represented in the panel. The pre-pandemic sera were not run with Roche ECLIA and Diasorin CLIA, and 100% specificity was assumed for both Roche ECLIA as stated by the manufacturer, and Diasorin CLIA as recently reported^9^. The main findings of the current study can be summarized as follows. First, direct comparison between IgG-FCI and Diasorin CLIA is biologically straightforward since both assays employ native SARS-CoV-2 S protein as the binding antigen and target the same antibody isotype. Notwithstanding this, both PPA and NPA were below 92% and inter-rater agreement between immunoassays was only moderate (k = 0.69). The lack of full concordance between the results provided by the two assays may relate to subtle differences in the conformation of the binding S protein: whereas in Diasorin CLIA the S protein bound to solid phase exhibits a stable native trimeric conformation, both trimeric and monomeric versions of the S protein were found to be displayed on the surface of transfected Jurkat T cells^12^. Furthermore, since SARS-CoV-2-S IgA responses can be documented in the absence of detectable SARS-CoV-2-S IgGs^18^, it was not unexpected to observe that PPA decreased whereas NPA increased when IgA FCI results were considered for the analyses, either individually or in combination with IgG ones. Second, despite the fact that Roche ECLIA measures total antibodies (IgG and IgM) binding to the RBD domain of S1 subunit protein instead of the native full-length S protein, we found excellent PPA between the results returned by this assay and by FCI (IgG, IgA or IgG/IgA), ranging between 96.1 and 97.7%, and strong inter-rater agreement (*k* value > 0.8), reinforcing the idea that humoral immune response against SARS-CoV-2 following natural infection is mainly directed towards RBD^3–5^. In turn, the lower NPA between FCI and Roche ECLIA than between FCI and Diasorin CLIA can be explained by the fact that highly immunogenic B-specific epitopes lie outside the RBD^3^. Third, overall both IgA and IgG/IgA-FCI returned more positive results overall than Roche ECLIA and Diasorin CLIA; However, this ultimately depended upon the time frame of serum collection after the onset of COVID-19 symptoms; in this sense FCI yielded more positive results than the other two immunoassays in early sera (drawn within 15 days after the onset of symptoms, whereas the Roche assay did so in sera collected afterwards. Interestingly, all three assays, most notably FCI, returned a number of positive results in sera that had scored negative by CLIA assays targeting recombinant S1/S2 subunit proteins or RBD, which were in use for routine diagnosis of SARS-CoV-2 infection at the time of testing request^19^. Assuming a specificity of 100% for all assays, these data suggest that the immunoassays evaluated herein, most strikingly FCI, may increase clinical sensitivity of previously marketed assays such as Liaison SARS-CoV-2 S1/S2 IgG CLIA and Maglumi 2019-nCoV IgG.

In our view, the main limitations of the current study are the relative small number of specimens included in the evaluation panel and that discrepancies across qualitative results returned by the evaluated immunoassays were not resolved by performing antibody neutralization assays, the gold standard for serological diagnosis of SARS-CoV-2 infection^6^. We also acknowledge as a limitation the fact that asymptomatic SARS-CoV-2 infected subjects were not included in the study. Thus, our conclusions only apply to acute or recovered COVID-19 individuals.

In summary, herein we have shown that a FCI using Jurkat T cells expressing the SARS-CoV-2 native S protein for detection of IgG and IgA-specific antibodies is highly specific and seemingly provides increased clinical sensitivity for diagnosis of SARS-CoV-2 infection when compared to two new-generation immunoassays targeting either the S protein in its trimeric conformation (Diasorin CLIA) or RBD (Roche ECLIA). The assay is easy to perform and standardize; the need for a flow cytometer should not be viewed as a disadvantage compared to high-throughput CLIA assays, as this platform is widely available at immunology and hematology departments in hospitals of all sizes. Further studies evaluating the performance of FCI for documenting seroconversion in vaccinated people are underway.

## Data Availability

The datasets generated during and/or analysed during the current study are available from the corresponding author on reasonable request.
